# Supplementary material for: Enhanced Luminance of CdSe/ZnS Quantum Dots Light-Emitting Diodes Using ZnO-Oleic Acid/ZnO Quantum Dots Double Electron Transport Layer
Source: Nanomaterials (Basel). 2022 Jun 14;12(12):2038. doi: 10.3390/nano12122038 (PMC9231060; doi:10.3390/nano12122038)
Supplement: Supplementary file 1 [file nanomaterials-12-02038-s001.zip › nanomaterials-1734110-supplementary.pdf]

Enhanced Luminance of CdSe/ZnS Quantum Dots Light-Emitting Diodes Using ZnO-Oleic Acid/ZnO Quantum Dots Double Electron Transport Layer

Da Young Lee [1,2,†], Hong Hee Kim [1,†], Ji-Hyun Noh [1], Keun-Yong Lim [1], Donghee Park [1,*], In-Hwan Lee [2], and Won Kook Choi [1,3,*]

[1] Center for Optoelectronic Materials and Devices, Korea Institute of Science and Technology, Seoul 02792, Korea; dayoung5178@naver.com (D.Y.L.); hongheekim85@kist.re.kr (H.H.K.); luzhexian@hanyang.ac.kr (J.-H.N.); t14822@kist.re.kr (K.-Y.L.)

[2] Department of Materials Science and Engineering, Korea University, Seoul 02841, Korea; ihlee@korea.ac.kr

[3] Department of Nanoscience and Engineering, KIST School, University of Science and Technology, Daejeon 34113, Korea

* Correspondence: pdmtime@kist.re.kr (D.P.); wkchoi@kist.re.kr (W.K.C.)

† These authors contributed equally to this work.

Figure S1. Deconvoluted Gaussian sub-peaks of O1s XPS spectra of (a) ZnO, (b) ZnO-OA (0.5 ml) and (c) ZnO-OA (1.0 ml), respectively.

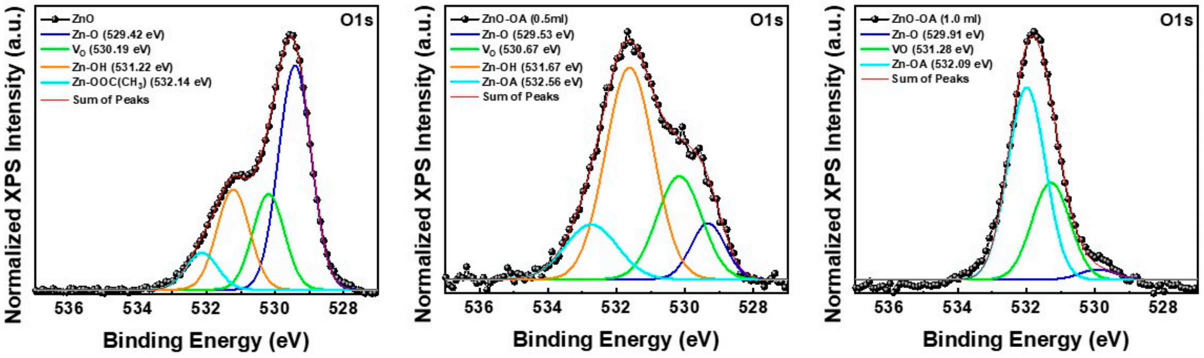

Calculated values: deconvoluted Gaussian sub-peaks of O1s XPS spectra of (a) ZnO, (b) ZnO-OA (0.5 ml) and (c) ZnO-OA (1.0 ml), respectively.

Model	Gauss			
Equation	y=y0 + (A/(w*sqrt(pi/2)))*exp(-2*((x-xc)/w)^2)			
Plot	Peak1(B)	Peak2(B)	Peak3(B)	Peak4(B)
y0	0.01725 ± 0.00115	0.01725 ± 0.00115	0.01725 ± 0.00115	0.01725 ± 0.00115
xc	529.42337 ± 0.16294	530.19252 ± 0.21216	531.22432 ± 0.13661	532.14024 ± 0.48295
w	0.9553 ± 0.08965	0.92296 ± 0.63664	0.95249 ± 0.54001	0.92095 ± 0.31084
A	1.02263 ± 0.47114	0.42045 ± 0.726	0.45425 ± 0.48341	0.16199 ± 0.21894
Reduced Chi-Sqr	1.26673E-4			
R-Square (COD)	0.99821			
Adj. R-Square	0.99806			

Model	Gauss			
Equation	y=y0 + (A/(w*sqrt(pi/2)))*exp(-2*((x-xc)/w)^2)			
Plot	Peak1(B)	Peak2(B)	Peak3(B)	Peak4(B)
y0	0.05353 ± 0.0026	0.05353 ± 0.0026	0.05353 ± 0.0026	0.05353 ± 0.0026
xc	529.32705 ± 1.21473	530.16701 ± 1.50183	531.62031 ± 0.38263	532.73915 ± 3.21811
w	0.99468 ± 1.27872	1.30824 ± 4.65014	1.4041 ± 1.45411	1.53027 ± 1.62373
A	0.26665 ± 2.92124	0.5455 ± 4.68947	1.42428 ± 4.00524	0.40037 ± 2.30771
Reduced Chi-Sqr	5.14493E-4			
R-Square (COD)	0.99411			
Adj. R-Square	0.9936			

Model	Gauss		
Equation	y=y0 + (A/(w*sqrt(pi/2)))*exp(-2*((x-xc)/w)^2)		
Plot	Peak1(B)	Peak2(B)	Peak3(B)
y0	0.05176 ± 0.00256	0.05176 ± 0.00256	0.05176 ± 0.00256
xc	529.91163 ± 1.4121	531.28659 ± 2.02117	532.09478 ± 0.88995
w	1.05431 ± 1.33975	1.10886 ± 1.52123	1.09282 ± 0.37244
A	0.04164 ± 0.14927	0.28578 ± 3.02394	1.04653 ± 2.90128+E
Reduced Chi-Sqr	6.48152E-4		
R-Square (COD)	0.98989		
Adj. R-Square	0.98925		

Table S1. **ZnO nanoparticles hybridized with various inorganic and organic materials for electron transport layer or emissive materials.**

	Materials	Approaches	Results	References
1	ZnO/OA	Passivation of surface defects	Strong blue emission	J. Am. Chem. Soc. **2007**, 129, 16029–16033
2	ZnO@TiO_2	Blue emitter	White emission	Sci. Rep. **2015**, 4, 4085−4090
3	$Zn_{1-x}Mg_xO$ (x=0~0.1)	ETL for QLEDs	Increased luminescence of CIS/ZnS QLEDs	Chem. Mater. **2015**, 27, 197–204
4	ZnO/PEIE	ETL for inverted QLEDs	Enhanced electron injection by lowering work function	Sci. Rep. **2015**, 5, 1–5
5	ZnO/OA	ETL for $MAObBr_3$-PVP PeLEDs*	Enhanced luminescence and current efficiency	J. Alloys Comp. 2019, 786, 11–17
6	ZnO@SnO_2	ETL for	Improved Efficiency of Perovskite Solar Cell	J. Am. Chem. Soc. **2019**, 141, 17610–17616
7	ZnO(Ga, Mg)	ETL for QLEDs	Optimization of e/h injection	RSC Adv. **2019**, 9, 32066–32071
8	ZnO/C_{60}	ETL for $CsPbI_3$ PeLEDs*	Enhanced luminescence by suppression of exciton quenching	J. Phys. Chem. C **2020**, 124, 28277–28284
9	ZnO-OA/ZnO	Double ETL for QLEDs	Enhanced luminescence by hole blocking	This study

*PeLEDs : perovskite LEDs

Table S2. XPS O1s spectra evaluated from ZnO, ZnO-OA (0.5ml) and ZnO-OA (1.0ml), respectively.

	Peak 1 (Zn-O)		Peak 2 (VO)	Peak 3 (Zn-OH)	Peak 4 (Zn-OOC) or (Zn-OA)	
ZnO	49.7		20.5	22.0	7.8	100
ZnO-OA (0.5ml)	9.9		20.6	54.2	15.3	100
ZnO-OA (1.0ml)	2.9		20.6		76.5	100
